# Protocol to assess engulfment and degradation of synaptosomes by murine microglia *in vitro*

**DOI:** 10.1016/j.xpro.2025.103936

**Published:** 2025-07-10

**Authors:** Alessandro Matera, Anne-Claire Compagnion, Rosa Chiara Paolicelli

**Affiliations:** 1 University of Lausanne, Department of Biomedical Sciences, 1005 Lausanne, Switzerland

**Keywords:** Cell culture, Microscopy, Neuroscience

## Abstract

Microglia, the innate immune cells of the central nervous system, refine neuronal circuitries both during brain development and in neurodegenerative diseases. Here, we present a protocol to independently assess the engulfment and degradation of synaptosomes by murine microglia, both in fixed and live samples, using confocal imaging. We describe steps for isolating primary microglia, preparing and conjugating synaptosomes with pHrodo, and performing an uptake and degradation assay. We then detail procedures for analyzing synaptosome uptake and degradation using ImageJ software.

For complete details on the use and execution of this protocol, please refer to Matera, Compagnion, et al.^1^

## Highlights

• Independent assessment of cargo uptake and degradation in primary microglia
• Preparation of primary microglia cultures and pHrodo-labeled synaptosomes
• Phagocytic assays using pHrodo-labeled synaptosomes in fixed and live microglia
• Quantification of engulfment and cargo degradation per cell using imaging analysis

## Abstract (editor highlights)

Publisher’s note: Undertaking any experimental protocol requires adherence to local institutional guidelines for laboratory safety and ethics.

## Graphical abstract

**Figure:**
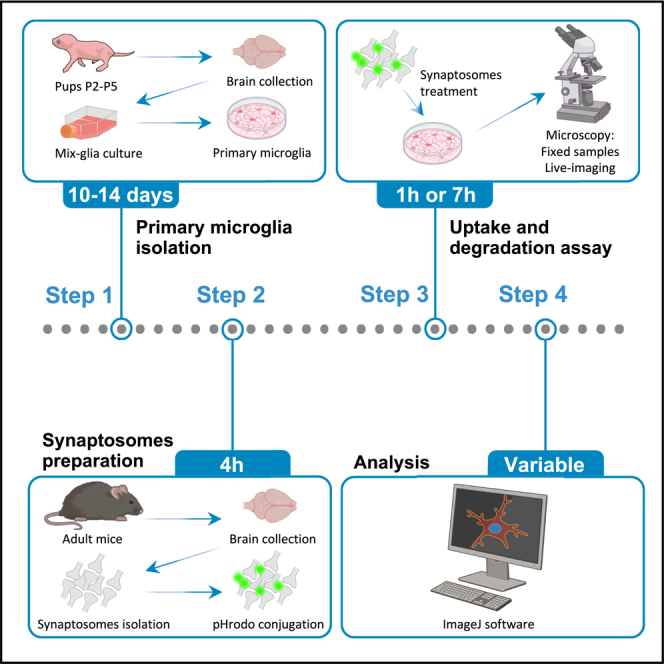

## Before you begin

Microglia play important roles in synapse homeostasis, contributing to the elimination of excess synapses during development.^2^^,^^3^^,^^4^^,^^5^^,^^1^ Thus, dysregulation of synaptic pruning could lead to defective brain wiring, with long-lasting consequences for brain function and behavior.^1^^,^^4^^,^^6^ Notably, microglia-mediated synapse refinement is also often imbalanced in several brain pathologies, such as schizophrenia and Alzheimer’s disease.^4^^,^^7^^,^^8^^,^^9^^,^^10^ A better understanding of the molecular mechanisms regulating this process may provide a promising target for future therapeutic strategies.

This protocol was designed to investigate the role of INPP5D, a gene whose risk variants have been associated with Alzheimer’s disease, in modulating microglial phagocytosis. Specifically, this protocol was used to assess how primary microglia KO for INPP5D differentially engulf and degrade fluorescently labeled-amyloid-β peptides or pHrodo-labeled synaptosomes when compared to control microglia (wild-type for INPP5D).^1^

The relevance of this protocol lies in its ability to discriminate between engulfment and degradation of the cargoes, two fundamental phases of phagocytosis.^11^^,^^12^ In fact, it is important to note that, at a given time point, an increase in the intracellular phagocytic cargo may derive either from an enhanced engulfment or from a defective degradation. Thus, it is critical to assess the two processes separately, to support meaningful and accurate data interpretation.

Microglia primary culture can be prepared from wild-type mice or from genetically modified mouse models to compare the impact of specific genes of interest. Additionally, the protocol enables the assessment of various pharmacological treatments, thus serving as a handy approach for compound screening. The methods detailed below describe the use of primary cells, but immortalized cell lines or human iPSC-derived microglia can also be used.

Furthermore, synaptosomes can be prepared from wild-type or genetically modified/disease mouse models, as well as from specific brain regions, allowing for a targeted evaluation of how synaptosome composition can influence microglial phagocytosis. Of note, sex differences can be considered when preparing primary microglia cultures or synaptosomes according to the experimental purpose.

Finally, this protocol can be adapted to diverse experimental needs, including cargo specificity and incubation duration. Overall, it provides a versatile tool for independently assessing microglial engulfment and degradation of various cargos.

### Institutional permissions

All animal experiments were authorized by the ‘Service de la consommation et des Affaires vétérinaires’ (SCAV) of the Canton de Vaud in Switzerland. Mice were purchased from The Jackson laboratory (on C57BL/6J background). Experiments were carried out on male and female animals. Mice were housed in groups and kept on a standard 12-h light/12-h dark cycle. All experiments were performed during the light cycle.

***Note:*** To use this protocol, it is mandatory to have permission for animal experiments from your relevant institution.

### Primary microglia culture

**Timing: 10–14 days**

In this section, we describe how to obtain primary microglia from the brain of C57BL/6J pups at postnatal day(P)2-P5. Briefly, after removing the olfactory bulb, the cerebellum, and the meninges from the brain, a mix-glia culture is obtained by enzymatic and mechanical dissociation. Finally, microglia cells can be harvested after 10–14 days and seeded for downstream experiments.

***Note:*** Materials required **-** HBSS, no calcium, no magnesium, no phenol red (Thermo fisher Scientific, Cat# 14175129); TrypLE Express Enzyme (1X), no phenol red (Thermo Fisher Scientific, Cat# 12604021); DMEM, high glucose, pyruvate (Thermo Fisher Scientific, Cat# 11995073); poly-D-Lysine hydrobromide – (Sigma-Aldrich, Cat# P7886); FBS (various); penicillin-Streptomycin (Thermo Fisher Scientific, Cat# 15140122); T75 flask with ventilated cap (Corning, Cat# 430641U); dissection tools (recommended: two forceps Dumont no. 5); μ-Slide 18 Well Glass Bottom (Ibidi, Cat# 81817) or 5 mm cover slides placed in 96-well plates; stereomicroscope (various); laminar hood.

#### Before starting

**Timing: 1.5 h for six pups**

1. Prepare culture media: DMEM high glucose (4.5 g/L) + 10% FBS + 1% Penicillin-Streptomycin and pre-warm at 37°C.
2. Prepare 15 mL tubes (one/brain) with 3 mL TrypLE Express Enzyme (1X), No Phenol Red and pre-warm at 37°C.
3. Place the dissection tools, Petri dishes (one per pup), and stereomicroscope under the laminar hood and turn on the UV for 15 min.

#### Brain collection

4. Sacrifice the pups by quick decapitation using sterilized scissors.
5. Dissect the brain and place it in HBSS 1x (3 mL) in a 15 mL tube on ice.
6. When all the brains have been collected (into separate tubes, on ice), move under a laminar hood.

#### Meninges removal

**CRITICAL:** From this point, everything needs to be done under a laminar hood to prevent contamination.

7. Move the brain in a Petri dish filled with cold HBSS 1x and place it under the stereomicroscope.
8. Discard the cerebellum and the olfactory bulb (using two forceps).
9. Remove the meninges as much as possible.

***Note:*** Using two sharp forceps, pinch the meninges and peel them from the brain. For more details on this procedure, see Güler et al.^13^

10. Transfer the leftover of the brain in HBSS 1x on ice.
11. Dice it with the forceps in 4–5 smaller pieces.
12. Process all brains before proceeding to the next steps.

#### Enzymatic and mechanical tissue dissociation and plating

13. Transfer each brain (all the small pieces) into a tube containing 3 mL of TrypLE Express Enzyme (pre-warmed up at 37°C).
14. Incubate for 20 min at 37°C (after the first 10 min, dissociate the samples with a 5 mL pipette, gently aspiring and withdrawing around 15 times/brain, and let incubate for the remaining 10 min).
15. After the enzymatic digestion, complete the dissociation gently aspiring and withdrawing with a P1000 (around 15 times).

***Note:*** At this point, the solution should look homogeneous with no visible big clumps.

16. Add 10 mL of warm culture media (final volume around 13 mL).
17. Spin down at 400 x g for 4 min at room temperature (RT).
18. Remove the supernatant and resuspend the pellet in 10 mL of warm culture medium.
19. Plate in T75 flask (one brain/flask).

#### Maintenance of cultures

**Timing: 10–14 days**

20. Change the medium completely after 24 h with fresh culture medium.
21. Every 2–3 days, change half the media volume.

***Note:*** Microglia will start appearing as spheroid bright cells on top of the astrocytic layer.

#### Seeding

22. Coat a plate: put enough volume of Poly-D-Lysine Hydrobromide at 0.1 mg/mL (in PBS 1X) to cover the surface of the wells, for at least 1 h in an incubator at 37°C.

***Note:*** For example, put 80–100 μL in a well of either 96-well plate or μ-Slide 18 Well Glass Bottom slides.

23. Wash three times with sterile dH_2_O.

***Note:*** Let the plate dry under the laminar hood, without the lid, for at least 10 min.

***Note:*** This step can be done in advance as coated plates, kept sterile, can be stored for extended periods (e.g., several weeks). Once dry, wrap the plate with parafilm and store at 4°C.

24. Take the T75 flask containing the mix-glia culture and tap to detach microglia from the astrocytic layer. Process all the flasks before passing to the next step.

***Note:*** The tapping of the flask is performed with the palm of the hand, with one tap every 2 s for 3–5 min, paying attention to not produce bubbles. We suggest controlling the cells’ detachment under a microscope. Repeat the tapping procedure if microglia are still attached to the astrocytic layer.

25. Collect the supernatant in a tube.
26. Centrifuge at 350 x g for 5 min at RT.
27. Collect and keep the supernatant (Glia Conditioned media - GCM).

**CRITICAL:** Keep the GCM of the mix-glia culture (step 27) and use it for seeding microglia cells and all subsequent treatments/medium changes. GCM must be stored at 4°C.

28. Resuspend the microglia pellet from step 26 in 1 mL of GCM.
29. Count the cells using an appropriate method (e.g. hemocytometer, automated cell counter).
30. Seed the cells on Poly-D-Lysine Hydrobromide-coated cover slides or glass-bottom slides.

***Note:*** For uptake and degradation assay we recommend seeding 10 000 cells on 5 mm cover slides placed in 96-well plates (for fixed samples assay) or in μ-Slide 18 Well Glass Bottom slides (for live-imaging assay or fixed samples assay).

31. Change the media completely with fresh GCM every 2–3 days.

***Note:*** To avoid repeated heating of GCM, warm up only the required quantity for the change of medium.

***Note:*** Before starting an experiment, wait 48 h for microglia to fully recover from the seeding.

Primary microglia cells obtained using this protocol can be maintained in culture for about 2 weeks, and they are not proliferating.

***Note:*** The purity of the isolated primary microglia can be assessed by immunofluorescence (e.g. staining against P2Y12R -microglia marker-, or IBA1 -macrophage marker-). If the purity is not optimal, please refer to “troubleshooting” section, problem 3.

### Synaptosome preparation and pHrodo conjugation

**Timing: 4 h**

In this section, we report how to isolate synaptosomes from a mouse brain using the Syn-PER Synaptic Protein Extraction Reagent and following manufacturer’s instructions (Thermo Fisher Scientific, Cat# 87793). Furthermore, we provide details on how to conjugate the isolated synaptosomes with pHrodo, a pH-sensitive dye, to later assess phagocytosis *in vitro*.

***Note:*** Material required - Syn-PER Reagent (Thermo Fisher Scientific, Cat# 87793); ice-cold phosphate-buffered saline (PBS, Thermo Fisher Scientific, Cat# 10010-056): 0.1 M sodium phosphate, 0.15 M sodium chloride, pH 7.2; refrigerated bench-top microcentrifuge; 20 mL syringe and 26G needle; dissection tools; Dounce tissue grinder; EDTA-free protease, phosphatase inhibitors (Thermo Fisher Scientific, Cat# A32961); pHrodo Green or Red Amine-Reactive Labels (Life technologies, Cat# P36600, P36012); Pierce BCA Protein Assay Kits (Thermo Fisher Scientific, Cat# 23227); laminar hood.

#### Before starting

32. Place the Dounce tissue grinder on ice before use.
33. Add inhibitors to the Syn-PER Reagent (only to the amount being used for the procedure and not to the stock solution).
34. Resuspend the pHrodo Green or Red Amine-Reactive Labels vials.
  a. 500 μg vial of amine-reactive pHrodo Green STP ester in 75 μL of DMSO (8.9 mM).
  b. 1 mg vial of amine-reactive pHrodo Red NHS ester in 150 μL of DMSO (10.2 mM).

#### Synaptosome isolation

35. Inject adult mice (2–3 months old) with 150 mg/Kg pentobarbital sodium intraperitoneally.

***Note:*** Alternative methods of euthanasia can be used (e.g. isoflurane, ketamine).

36. Check for complete anesthetic state (e.g., loss of pain reflex by lack of movement of paw/tail when squeezed).
37. Perfuse the mice intracardially with 20 mL ice-cold PBS (flow rate of ∼10 mL per minute, for 2 month-old mice) using a 20 mL syringe.
38. Remove the brain and dissect the brain region of interest.

***Note:*** All the following steps should be performed under a laminar hood.

39. Weigh neuronal tissue samples and add 10 mL of Syn-PER Reagent per gram of tissue (e.g., 2 mL of Syn-PER Reagent per 200 mg of brain tissue).
40. Perform Dounce homogenization on ice with ∼10 slow strokes.
41. Transfer the homogenate to a 1.5 mL tube.
42. Centrifuge the tube at 1200 x *g* for 10 min at 4°C. Transfer the supernatant to a new tube and discard the pellet.
43. Centrifuge the supernatant at 15000 × *g* for 20 min at 4°C.
44. Remove the supernatant from the synaptosome pellet and discard.
45. Add 1–2 mL of Syn-PER reagent per gram of tissue sample, to suspend the synaptosome pellet (e.g., 500 μL for 200-400 mg of brain tissue).
46. Maintain the synaptosome suspension on ice and continue with the pHrodo conjugation protocol.

**Pause point:** Unlabeled synaptosome suspension can be stored in 5% (v/v) DMSO at −80°C or in liquid nitrogen for extended periods (for a maximum of 1 year).

***Note:*** The enrichment of synaptosomes fraction can be assessed by western blot assay, comparing the homogenate fraction (supernatant in the step 42) to the synaptosomes one (obtained at the end of the step 44). We suggest verifying the enrichment of both pre- and post-synaptic markers (e.g. vGLUT1, vGAT, HOMER1, PSD-95 and GEPHYRIN).

#### pHrodo conjugation using pHrodo green or red

47. Measure the protein concentration of synaptosome samples using the BCA kit according to the manufacturer’s instructions (the expected concentration range is between 1 and 2 mg/mL).
48. Calculate the amount of protein you want to label.
49. Centrifuge the synaptosome preparation at 15000 x g for 20 min, at 4°C.
50. Resuspend in 0.1 M sodium bicarbonate buffer, pH 8.4 to reach a concentration of 1 mg/mL.

***Note:*** For example, take the volume for 100 μg of synaptosome preparation (about 100 μL). After centrifugation, resuspend in 100 μL of sodium bicarbonate 0.1 M pH 8.4.

51. Add the appropriate amount of reactive dye to the protein solution and mix by pipetting up and down several times.

***Note:*** Dye-to-protein molar ratio should be 5–20 moles of dye per mole of protein.

***Note:*** For example, add 6 μL of pHrodo dye to 100 μg of synaptosome preparation.

52. Incubate the reaction for 60 min at RT, protected from light with small agitation.
53. Centrifuge at 15000 x g for 20 min at 4°C.
54. Resuspend the pellet in the same volume used in step 50 of Syn-Per Reagent (+inhibitors).

***Note:*** For example, add 100 μL to the pellet.

***Note:*** Unlabeled and pHrodo-labeled synaptosome suspension can be stored in 5% (v/v) DMSO at −80°C or in liquid nitrogen for extended periods.

## Key resources table

**Table:** 

REAGENT or RESOURCE	SOURCE	IDENTIFIER
**Chemicals, peptides, and recombinant proteins**		

Paraformaldehyde	Sigma-Aldrich	Cat# 16005
Poly-D-Lysin-hydrobromid	Sigma-Aldrich	Cat# P7886
Beta-Amyloid (1-40), HiLyte Fluor 488-labeled	Eurogentec	Cat# AS-60491-01
Dimethyl sulphoxide (DMSO)	Roth	Cat# A994.1
pHrodo iFL Green STP ester	Life Technologies	Cat# P36012
pHrodo Red, succinimidyl ester	Life Technologies	Cat# P36600
CytochalasinD	Sigma-Aldrich	Cat# C8273
Bafilomycin	Hello Bio	Cat# HB1125
Pierce Protease and Phosphatase Inhibitor Mini Tablets, EDTA-free	Thermo Fisher Scientific	Cat# A32961

**Critical commercial assays**		

Syn-PER Synaptic Protein Extraction Reagent	Thermo Fisher Scientific	Cat# 87793
Pierce BCA Protein Assay Kit	Thermo Fisher Scientific	Cat# 23227

**Experimental models: Organisms/strains**		

C57Bl/6J (P2–P5, 2–3 months old)	The Jackson Laboratory	Cat# 000664

**Software and algorithms**		

ImageJ	Schneider et al.^14^	RRID: SCR_003070
Stellaris 5 confocal laser scanning system	Leica	RRID: SCR_024663

**Other**		

TrypLE Express Enzyme (1X), no phenol red	Thermo Fisher Scientific	Cat# 12-604-021
PBS 1X	Thermo Fisher Scientific	Cat# 10010-056
DMEM, high glucose, pyruvate	Thermo Fisher Scientific	Cat# 11995073
Fetal bovine serum, qualified, heat inactivated, Brazil	Thermo Fisher Scientific	Cat# 10500064
Penicillin-Streptomycin (10,000 U/mL)	Thermo Fisher Scientific	Cat# 15140122
HBSS, no calcium, no magnesium, no phenol red	Thermo Fisher Scientific	Cat# 14175129
T75 flask with ventilated cap	Corning	Cat# 430641U
μ-Slide 18-well glass bottom	ibidi	Cat# 81817
ibidi Gas incubation system, CO_2_/O_2_	ibidi	Cat# 11923
ibidi Heating system slide/dish	ibidi	Cat# 12110

## Step-by-step method details

In this section, we report how to dissect two important phases of phagocytosis: uptake and degradation of a cargo. Specifically, we describe how to investigate microglia phagocytic capacity towards synaptosomes, *in vitro.* The assessment of these processes can be performed using either PFA-fixed samples or by live-imaging. In addition, we suggest experimental controls that should be implemented to validate the engulfment and the degradation investigated.

### Assessing synaptosome uptake by primary microglia in fixed samples

**Timing: 1 h 30 min**

These steps outline the treatment of primary microglia with pHrodo-labeled synaptosomes to evaluate their uptake in fixed samples.

***Note:*** Material required - Phosphate-buffered saline (PBS, Thermo Fisher Scientific, Cat# 10010-056): 0.1 M sodium phosphate, 0.15 M sodium chloride, pH 7.2; GCM: obtained during the primary microglia isolation; pHrodo-labeled synaptosomes; chemical hood; laminar hood; paraformaldehyde (PFA, Sigma-Aldrich, Cat# 16005); μ-Slide 18 Well Glass Bottom (Ibidi, Cat# 81817) or 5 mm cover slides placed in 96-well plates; mounting medium (various).

1. Pre-warm the GCM at 37°C.
2. Thaw pHrodo-labeled synaptosomes on ice and protect them from light.
3. Switch off the light in the laminar hood.
4. Dilute pHrodo-labeled synaptosomes at 1 μg/100 μL in GCM (Working solution: WS) and keep it covered from light.

***Note:*** For μ-Slide 18 Well Glass Bottom, 100 μL per well will be sufficient for the treatment.

***Note:*** Add one or two wells not treated with pHrodo-labeled synaptosomes as controls. As microglia display some autofluorescence, this condition will help using the correct settings for confocal acquisitions (negative control).

***Note:*** Make sure to prepare enough solution (prepare for one or two extra wells).

***Note:*** We have tested this protocol with other cargoes. E.g., with 1 μM fluorescently labeled-Amyloid ß 1-40 488 (Eurogentec, Cat# AS-60491-01) to assess Amyloid ß uptake.

5. Remove the culture media from the seeded primary microglia.
6. Add 100 μL of WS in each well.
7. Leave the plate in an incubator at 37°C and 5% CO_2_ for 1 h.

***Note:*** If the assay is performed with cells other than murine primary microglia and with different cargos, the timing of uptake may vary. Perform preliminary experiments to determine the appropriate temporal dynamics. Example**:** when using 1 μM Amyloid ß 1-40, incubate for 3 h.

Before treating the cells with the cargo of interest, proceed to a visual inspection, to assess viability and morphology. Cultures with abnormal morphology (e.g. round cells, should not be used for the experiment). See representative images for primary cells appearance in vitro.

8. At the end of the treatment, remove the WS from the well and wash three times with 100 μL PBS, without incubation time in between.

**CRITICAL:** Performing a careful wash of the wells is critical to remove free-floating and glass-attached synaptosomes, and synaptosomes that are attached to the cell surface but not engulfed by microglia.

9. Bring the plate under a chemical hood.
10. Remove PBS, add 100 μL of 4% PFA in each well.
11. Incubate for 20 min at RT. Keep the plate protected from light.
12. At the end of the incubation, remove the PFA from the well and wash three times with PBS.

***Note:*** Leave 100 μL of PBS in each well. The Ibidi plate is ready to be acquired. If the cells were seeded on 5 mm cover slides in 96-well plates, mount the cover slide (upside down) on a microscopy slide, with mounting medium, let dry overnight.

***Note:*** If needed, after fixation of the samples, immunofluorescence assay can be performed prior to mounting.

**CRITICAL:** We suggest acquiring the images as soon as possible to avoid sample contamination and loss of the pHrodo signal. To store the plate, seal it with parafilm, cover it with aluminum foil, and keep it in the fridge.

### Assessing synaptosome degradation by primary microglia in fixed samples

**Timing: 7 h**

These steps outline the treatment of primary microglia with pHrodo-labeled synaptosomes to evaluate their degradation in fixed samples.

***Note:*** Material required - Phosphate-buffered saline (PBS, Thermo Fisher Scientific, Cat# 10010-056): 0.1 M sodium phosphate, 0.15 M sodium chloride, pH 7.2; GCM: obtained during the primary microglia isolation; pHrodo-labeled synaptosomes; chemical hood; laminar hood; paraformaldehyde (PFA, Sigma-Aldrich, Cat# 16005); μ-Slide 18 Well Glass Bottom (Ibidi, Cat# 81817) or 5 mm cover slides placed in 96-well plates.

13. Pre-warm the GCM at 37°C.
14. Thaw pHrodo-labeled synaptosomes on ice and protect them from light.
15. Follow the protocol “Assessing synaptosomes uptake by primary microglia in fixed samples” until the point 8.
16. Add 100 μL of GCM in each well and place back the plate in an incubator at 37°C and 5% CO_2_ for additional 6 h.

***Note:*** It is critical to perform in parallel uptake and degradation using the same cell preparation and the same reagents (e.g. conjugated synaptosomes). While the plate designated for uptake assessment will be fixed and stored, the plate for degradation assessment will be further processed as detailed below.

***Note:*** If the assay is performed with cells other than murine primary microglia and with different cargos, the timing of the degradation may vary. Perform trial experiments to determine the appropriate temporal dynamics.

17. At the end of the incubation, remove GCM from the well and wash three times with PBS.
18. Bring the plate under a chemical hood and put 100 μL of PFA 4% in each well, for 20 min at RT. Keep the plate protected from light.
19. At the end of the incubation, remove the PFA from the well and wash three times with PBS. Leave 100 μL of PBS in each well.

***Note:*** The Ibidi plate is ready to be acquired. If the cells were seeded on 5 mm cover slides in 96-well plates, mount the cover slide (upside down) on a microscopy slide, with mounting medium, let dry overnight.

***Note:*** If needed, after fixation of the samples, immunofluorescence assay can be performed prior to mounting.

**CRITICAL:** We suggest acquiring the images as soon as possible to avoid contamination and loss of the signal. To store the plate, seal it with parafilm, cover it with aluminum foil, and keep it in the fridge.

### Assessing synaptosome uptake in primary microglia by live imaging

**Timing: 1 h 30 min**

These steps outline the treatment of primary microglia with pHrodo-labeled synaptosomes to evaluate their uptake through live-imaging.

***Note:*** Material required - Phosphate-buffered saline (PBS, Thermo Fisher Scientific, Cat# 10010-056): 0.1 M sodium phosphate, 0.15 M sodium chloride, pH 7.2; GCM: it is obtained during the primary microglia isolation; pHrodo-labeled synaptosomes; chemical hood; laminar hood; inverted fluorescence microscope suitable for live imaging (Confocal microscope optimally); humidity and temperature control, and CO2/O2 regulation chamber for live-imaging (Ibidi, Cat# 11923, #12110); μ-Slide 18 Well Glass Bottom (Ibidi, Cat# 81817).

20. Pre-warm the GCM at 37°C.
21. Thaw pHrodo-labeled synaptosomes on ice and protect them from light.
22. Set and switch on the humidity and temperature control chamber (Humidity 80% and Temperature 37°C, O_2_ 20%, CO_2_ 5%).
23. Switch on the fluorescence microscope.
24. Follow the protocol “Assessing synaptosomes uptake by primary microglia in fixed samples” until the point 5.
25. Add 100 μL of WS in each well and rapidly move the plate inside the humidity and temperature control chamber.
26. Choose the appropriate objective of the microscope, the timing of the acquisition, the number of frames, and the z stack.

***Note:*** Objective of the microscope: we recommend using a 20X/0.75 dry objective with digital zoom 2.0, as primary microglia are highly mobile, and they may move out from acquisition field of view over time. It is also suggested to perform a tile scanning. Use higher magnification objective to better appreciate the intracellular trafficking of the cargo.

***Note:*** Timing of the acquisition: for primary microglia we recommend acquiring for 1 h. However, if the assay is performed with cells other than primary microglia, the dynamic of cargo uptake may change. Perform trial experiments to evaluate the correct timing.

***Note:*** Number of frames: 15 min interval between each frame is sufficient to appreciate progressive increase of cargo engulfment over time.

***Note:*** Z stack: As primary microglia are highly mobile, the focal plane may rapidly change. Thus, although the correct z stack should be around 4-5 μm, we recommend acquiring a stack of 10 μm, with a 1 μm z-step, to ensure that the cells of interest remain in focus throughout the entire acquisition period. To shorten the acquisition time of a single z stack, the acquisition frequency can be increased (600–700 Hz), and bidirectional scanning mode can be used.

**CRITICAL:** Pay attention to the settings of the microscope. The intensity of the pHrodo signal will increase over time, due to the cargo trafficking to the lysosomes. Thus, avoid acquiring a close-to-saturation signal from the first frames.

***Note:*** Include in the experimental design one or two wells containing microglia not treated with pHrodo-labeled synaptosomes. As microglia display some autofluorescence, these wells will help adjusting the correct microscope settings (negative control).

***Optional:*** After the imaging, the plate can be removed from the humidity and temperature control chamber and the cells can be fixed for further immunostaining experiments. Then, follow the protocol “Assessing synaptosomes uptake by primary microglia in fixed samples” starting from point 8.

### Assessing synaptosome degradation in primary microglia by live imaging

**Timing: 7 h**

These steps outline the treatment of primary microglia with pHrodo-labeled synaptosomes to evaluate their degradation through live-imaging.

***Note:*** Material required - Phosphate-buffered saline (PBS, Thermo Fisher Scientific, Cat# 10010-056): 0.1 M sodium phosphate, 0.15 M sodium chloride, pH 7.2; GCM: it is obtained during the primary microglia isolation; pHrodo-labeled synaptosomes; chemical hood; laminar hood; inverted fluorescence microscope suitable for live imaging (Confocal microscope optimally); humidity and temperature control, and CO2/O2 regulation chamber for live imaging (Ibidi, Cat# 11923, #12110); μ-Slide 18 Well Glass Bottom (Ibidi, Cat# 81817).

27. Pre-warm the GCM at 37°C.
28. Thaw pHrodo-labeled synaptosomes on ice and protect them from light.
29. Set and switch on the humidity and temperature control chamber (humidity 80%, temperature 37°C, O_2_ 20%, CO_2_ 5%).
30. Switch on the fluorescence microscope.
31. Follow the protocol “Assessing synaptosomes uptake by primary microglia in fixed samples” until the point 8.
32. Add 100 μL of GCM in each well and rapidly move the plate inside the humidity and temperature control chamber.
33. Choose the appropriate objective of the microscope, the timing of the acquisition, the number of frames, and the z stack.

***Note:*** Objective of the microscope: we recommend using a 20X/0.75 dry objective with digital zoom 2.0, as primary microglia are highly mobile, and they may move out from acquisition field of view over time. It is also suggested to perform a tile scanning. Use higher magnification objective to better appreciate the intracellular trafficking of the cargo.

***Note:*** Timing of the acquisition: for primary microglia we recommend acquiring for at least 6 h. However, if the assay is performed with cells other than primary microglia, the timing of the degradation may change. Perform trial experiments to evaluate the appropriate temporal dynamics.

***Note:*** Number of frames: 1 h between each frame and the other is sufficient to appreciate cargo degradation.

***Note:*** Z stack: As primary microglia are highly mobile, the focal plane may rapidly change. Thus, despite the correct focal plane should be around 4-5 μm, we recommend acquiring a stack of 10 μm, with a 1 μm z-step, to ensure that the cells of interest remain in focus throughout the entire acquisition period.

**CRITICAL:** Pay attention to the settings of the microscope as the intensity of the pHrodo signal will decrease over time, due to progressive cargo degradation. We suggest setting the pHrodo signal just below saturation.

***Optional:*** After the acquisition, the plate can be removed from the humidity and temperature control chamber and the cells can be fixed for further immunostaining experiments. Then, follow the protocol “Assessing synaptosomes degradation by primary microglia in fixed samples” starting from point 17.

### Experimental controls for phagocytic assays

These steps outline experimental controls to validate the accuracy of the pHrodo signal, ensuring it is distinguishable from background noise (with Bafilomycin A1 treatment), and to evaluate the phagocytosis component (with Cytochalasin D treatment).

***Note:*** Material required - Cytochalasin D (Sigma-Aldrich, Cat# C8273): widely used inhibitor of actin polymerization, required for phagocytic processes. Dissolve Cytochalasin D in DMSO, following the manufacturer’s instructions; Bafilomycin A1 (Hello Bio, Cat# HB1125): widely used inhibitor of V-ATPase, required for lysosomal acidification. Dissolve Bafilomycin in DMSO, following the manufacturer’s instructions.

34. Inhibit cargo uptake:
  a. Pre-treat the cells with Cytochalasin D, diluted at 5 μM in GCM, for 30 min.
    ***Note:*** Keep the cells in an incubator at 37°C and 5% CO_2,_ during the incubation.
  b. Follow the protocol “Assessing synaptosomes uptake in primary microglia in fixed samples” or “Assessing synaptosomes uptake in primary microglia by live-imaging”.
    ***Note:*** It is required to maintain the cells under Cytochalasin D treatment during the synaptosome incubation, to constantly prevent phagocytic events.
35. Assess the specificity of the pHrodo signal:
  a. Pre-treat the cells with Bafilomycin A1, diluted at 100 nM in GCM, for 30 min.
    ***Note:*** Keep the cells in an incubator at 37°C and 5% CO_2,_ during the incubation.
  b. Follow the protocol “Assessing synaptosomes uptake in primary microglia in fixed samples” or “Assessing synaptosomes uptake in primary microglia by live-imaging”.
    ***Note:*** It is required to maintain the cells under Bafilomycin treatment during the synaptosome incubation, to constantly prevent the acidification of the lysosomes.
    ***Note:*** Bafilomycin A1 treatment can also be used to block lysosomal degradation. In this case, incubate the cells with 100 nM Bafilomycin A1 only after the incubation with synaptosomes. Incubate the cells for the whole time of degradation (6 h). Inhibition of lysosomal acidification results in defective degradation of the cargo, compared to DMSO controls.

For these experiments, it is important to use fluorescently-labeled synaptosomes (e.g., TdTomato-synaptosomes) whose fluorescence is not depending on the pH. These synaptosome can be isolated from genetically modified mouse models with neurons expressing a reporter fluorescent protein (e.g. CAMKII^cre^;Rosa26-TdTomato^fl^).

Alternatively, the internalized cargo can be detected upon fixation by immunostaining.

### Analysis of synaptosome uptake and degradation using ImageJ software

The proposed analysis requires the ImageJ software^14^ and renders the area covered by pHrodo-labeled synaptosomes per cell. Image analysis should be performed blind to the experimenter, for reducing human bias (e.g., modifying the name of the images with randomized coding numbers).

36. Select Region of Interest (ROI):
  a. Open the image with ImageJ.
  b. Using the bright field (or a specific microglial cytoplasmic marker -e.g. IBA1- or reporter mouse line -e.g. Cx3cr1EGFP- to visualize the individual cells) draw a ROI around each cell with one of the drawing tools (“free-hand” or “polygon” selection in the toolbar).
  c. After drawing the contour of one cell, add it to the ROI manager: Edit→ Selection→ Add to manager (shortcut: Ctrl+T). A table should appear with the selected area.
  d. Repeat passage “c” for all the cells in the field of view.
  e. After all the cells are drawn, save the ROIs to a new folder, called as the name of the image. ROI Manager → More → Save.
    **CRITICAL:** Make sure to select all the ROIs before saving, otherwise only the first ROI will be saved.
  f. Repeat for all the images that need to be analyzed.
37. Select proper threshold:
  ***Note:*** You will need to define a unique threshold for your signal of interest (e.g. pHrodo-labeled synaptosomes) to be able to measure and compare the area covered by your signal among the cells. To do this, the threshold should be optimally defined to fit the different conditions of the experiment.
  **CRITICAL:** Both uptake **AND** degradation, acquired with the same settings, need to be analyzed using the same threshold across the different conditions if you want to compare them.
  a. Make sure to set a threshold that will fit your different conditions (Figure S1).
  b. Open the image(s) with ImageJ.
  c. If stacks were acquired, Z stack project the image: Image → Stacks → Z Project, projection type: Max intensity.
  d. On the channel of interest, set the correct threshold: Image → Adjust → Threshold (shortcut: Ctrl+Shift+T). Select dark background and change the minimal value to highlight the area you want to analyze. After setting the correct threshold, click apply.
    **CRITICAL:** Here, you will define the optimal threshold using several representative images. After defining it, note it down and make sure to use the same for all the images.
  e. Set the background to Dark.
  f. Tick “Black background (of binary masks)”. The image will be changed to a binary black and white image.
    **CRITICAL:** Check that the Method set is the same you used to define the threshold.
38. Measure the signal:
  a. Set the options you want to measure: Analyze → Set Measurements. Select at least Area fraction.
    ***Note:*** Area fraction will measure the percentage of the area covered by your signal within each individual cell.
  b. Open the ROI manager: Analyze → Tools → ROI Manager.
  c. Open the ROIs you previously defined for the selected image: ROI Manager → More → Open.
  d. Select all the ROIs and click on measure. ROI Manager → Measure.
  e. A table with the measurements will pop out. Save the table with the appropriate file name.
  f. Repeat for all the images that you need to analyze, making sure to use the same threshold.
    ***Note:*** For live-imaging experiments, repeat the ROI selection for each timeframe.
    ***Note:*** This analysis will give you the area covered by your signal per cell.
39. Data representation and interpretation:
  a. Plot the average value of the area covered by your signal per cell, per each independent replicate.

***Note:*** Data should be analyzed by the average area covered over independent experiments. In addition, the cell distribution can be displayed per condition within each experiment.

***Note:*** For a proper experiment, we recommend acquiring multiple fields of view from 2/3 technical replicates (wells) over 3/4 independent experiments (independent microglia preparations).

***Note:*** For fixed and live imaging experiments, data can be represented as: uptake, residual levels, and efficiency of degradation (see expected outcomes).

## Expected outcomes

### Primary microglia

**Figure 1:**
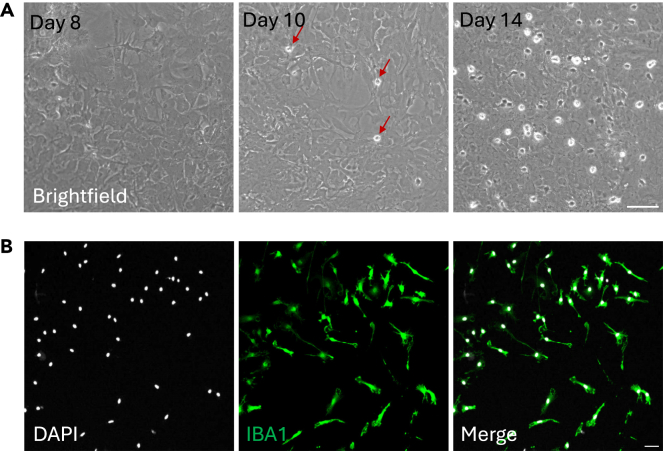
Gradual emergence of microglia from mix-glia culture and microglia purity after seeding (A) Left: Representative image of mix glia culture – astrocytic layer- before microglia rise. Middle: Few microglia appear round and bright on top on the astrocytic layer (indicated with red arrows). Right: Mix glia culture with microglia at an optimal time-point for collection (DIV14). Scale bar 100 μm. (B) Micrographs of IBA1 staining after primary microglia seeding on poly-D-lysine coated-cover slips. All cells should be IBA1+. Scale bar: 15 μm.

The estimated time to harvest microglia cells is about 10–14 days, and 300 000 - 500 000 cells are expected to be collected per brain. They will grow on top of the astrocytic layer and appear as small round bright cells (Figure 1A). Following this protocol, the purity of microglia cells after seeding is around 99% (Figure 1B). However, please confirm the purity of the culture when establishing the protocol, using an IBA1 staining. Please, note that IBA1 is a microglia/macrophage marker. For further characterization, P2Y12R, TMEM119 and HEXB could be used as additional markers to identify microglia.

### Synaptosome preparation

The expected concentration of synaptosomes isolated is between 1 and 2 mg/mL.

### Synaptosome uptake and degradation

**Figure 2:**
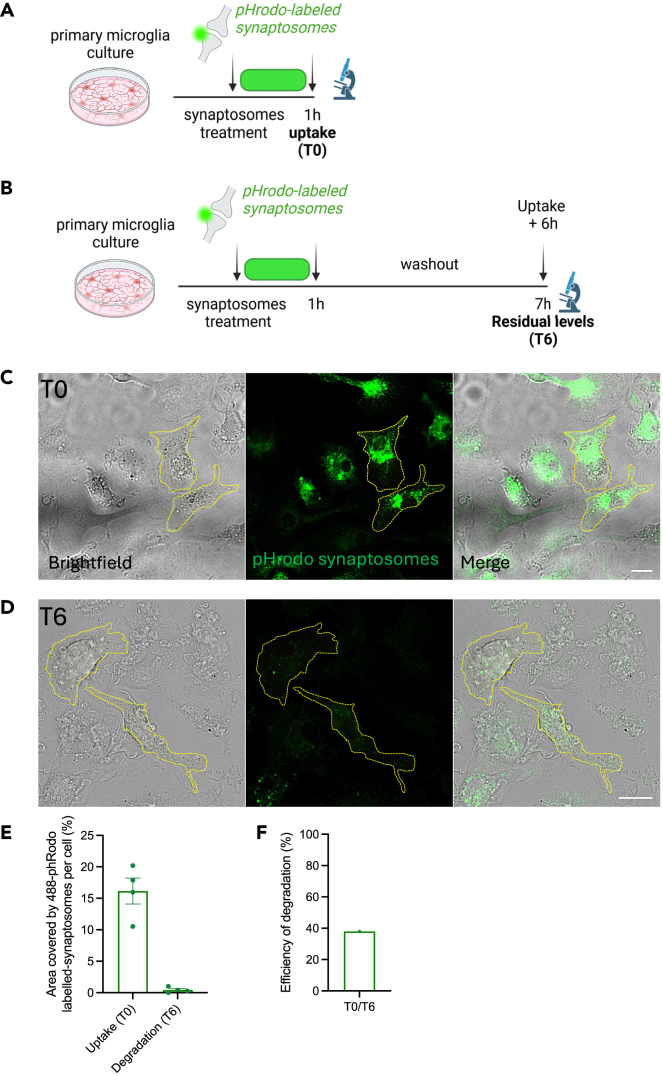
Experimental scheme of uptake and degradation of pHrodo-labeled synaptosomes on fixed cells (A) Experimental design of the uptake assay. (B) Experimental design to assess degradation after cargo uptake. This assay has to be done in parallel with the uptake assay. (C) Representative z stack confocal acquisition of 488-pHrodo-labeled synaptosomes engulfed by primary microglia after 1 h treatment. Scale bar: 15 μm. (D) Representative confocal image showing residual synaptosomal signals within microglia 6 h post wash out. Scale bar: 15 μm. (E) Quantification of the area covered by 488-pHrodo-labeled synaptosomes per cell (%) of C (T0=uptake) and D (T6=degradation). Dotted lines are an example of cell contouring. Data are represented as mean ± SEM. Each dot represents a cell. Please, note that this quantification is for explanatory purposes only: multiple fields of view should be imaged per condition, and independent biological experiments should be performed. (F) Quantification of efficiency of degradation (T0/T6). *Adapted from Matera, Compagnion et al., Immunity, 2025.*

**Figure 3:**
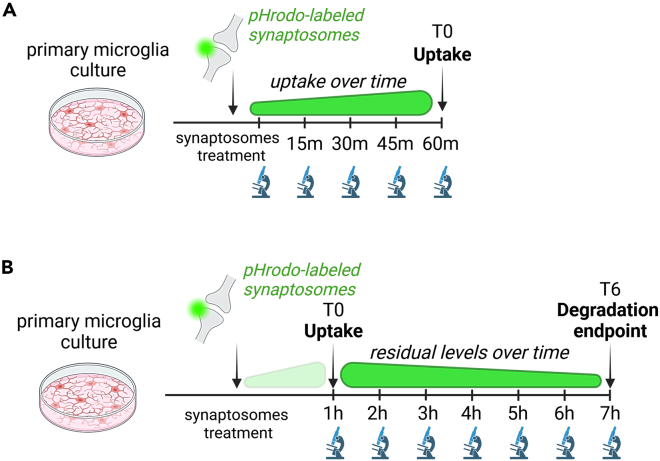
Experimental scheme to assess uptake and degradation of pHrodo-labeled synaptosomes in live cells (A) Scheme of the experimental uptake assay. (B) Scheme of the experimental assay to assess degradation capacity after cargo uptake. *Adapted from Matera, Compagnion et al., Immunity, 2025*.

It is expected to observe an increase in the pHrodo signal when assessing the synaptosome uptake after 1 h (T0) -for fixed samples: Figures 2A–2C; for live imaging: Figure 3A; Methods video S1: Representative video of green pHrodo-labeled synaptosomes uptake by microglia-; and a decreased signal when evaluating synaptosome degradation after 6 h post washout (T6) -for fixed samples: Figures 2B–2D; for live imaging: Figure 3B; Methods video S2: Representative video of green pHrodo-labeled synaptosomes degradation by microglia-. The parameters that can be quantified are: Uptake (T0: quantity of cargo engulfed after a given amount of time); residual levels (Degradation=T6: quantity of cargo remaining in the cell after the degradation period -after wash out of the cargo-); and efficiency of degradation (Index of degradation efficiency for a given cargo over a given time, calculated as the amount of cargo uptaken -e.g. 1 h after synaptosome incubation- divided by the residual amount measured at the end of the degradation experiment -6 h after wash out-). *Efficiency of degradation = Uptaken cargo / Residual levels* -see Figures 2C–2F for representative images and relative analysis-.

Methods video S1. Representative video of green pHrodo-labeled synaptosomes uptake by microglia, related to step-by-step method details, step 26 Scale bar: 50 μm. Speed: 3 fps.

Methods video S2. Representative video of green pHrodo-labeled synaptosomes degradation by primary microglia, related to step-by-step method details, step 33 Scale bar: 50 μm. Speed: 3 fps.

**Figure 4:**
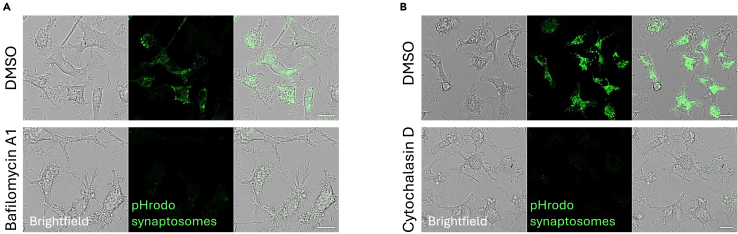
Uptake of pHrodo-labeled synaptosomes in fixed cells with or without Bafilomycin A1 or Cytochalasin D treatment (A) Representative z stack confocal acquisition of 488-pHrodo-labeled synaptosome engulfed by primary microglia with or without Bafilomycin A1. Scale bar: 15 μm. (B) Representative z stack confocal acquisition of 488-pHrodo-labeled synaptosome engulfed by primary microglia with or without Cytochalasin D. Scale bar: 15 μm. *Adapted from Matera, Compagnion et al., Immunity, 2025*.

The pHrodo signal is expected to be undetectable when Bafilomycin A1 or Cytochalasin D are applied to the cells, as lysosomal acidification or actin-mediated phagocytosis are prevented by these treatments, respectively (Figures 4A and 4B).

### Analysis

**Figure 5:**
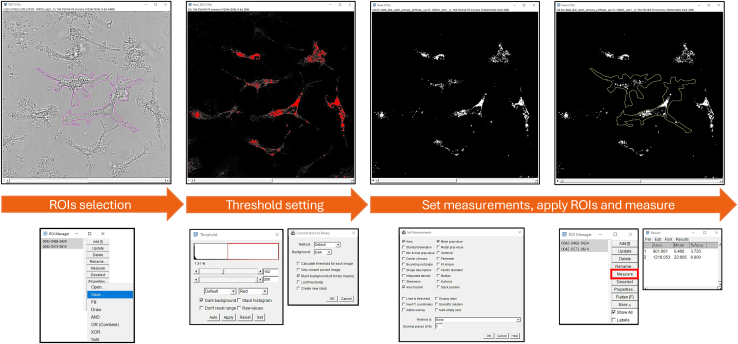
Workflow for the analysis of the area covered by pHrodo signal within microglia After drawing the ROIs contouring the cells of interest, a threshold is applied, allowing the measurement of the area covered by the signal per individual cell.

The analysis described in this protocol is expected to work for any labeled and engulfed cargo and for any cells whose profile can be drawn (Figure 5).

## Limitations

### Primary microglia

As this protocol requires an initial step of mix-glia culture, the timing and yield of primary microglia may change if genetically modified mice are used. Genetical mutations may indeed affect the initial proliferation and survival of microglia, as well as factors contained in the GCM. Furthermore, microglia cells are not in a proliferative state after seeding. Thus, the quantity of pups to be used should be defined according to the final number of cells needed for the experimental plan.

### GCM composition

When culturing primary microglia, we use glial-conditioned media to support their survival, as it provides essential growth factors. However, this can introduce potential bias. Variability in the composition of the GCM between experiments can affect reproducibility, as slight differences in growth factor concentrations or other components could influence microglial responses. Additionally, it should be considered that GCM composition could differ according to the diverse treatments or genotypes to be compared, and that soluble factors contained in the GCM could influence the phagocytic capacity of microglia. Alternatively, the GCM from different conditions (e.g. different genotypes) could be mixed at the time of microglial harvesting, to reduced potential variability.

GCM could be replaced with defined-medium composition, as previously described in Bohlen et al., 2017.^15^

### Neonatal versus adult microglia

Microglial phagocytosis plays diverse roles throughout development and disease. In neonatal mice, microglia are essential for scavanging apoptotic neurons, pruning synapses, and shaping neural plasticity. In adults, phagocytic activity of microglia is key to ensure proper immune surveillance in physiological states, and becomes prominent in a wide range of pathological conditions, from traumatic brain injuries, to infection and neurodegenerative diseases.

Since this model uses neonatal microglia, it better recapitulates early microglial states, which could be linked to functional assays relevant in early developmental stages.

To better model microglial functions in adult or neurodegenerative conditions, primary microglia can be isolated from older mice. Several protocols are available.^16^^,^^17^ However, isolating microglia from adult or aged mice presents challenges, including lower cell yield, increased cell stress, and altered microglial phenotypes. Additionally, the presence of myelin debris complicates purification, often requiring additional steps such as myelin removal columns, making the protocol more labor-intensive and technically demanding.

Despite these challenges, using adult microglia, ideally isolated directly from models of pathology and related healthy controls, could provide more relevant approaches for studying neurodegenerative disease mechanisms.

### Mouse versus human microglia

Although this protocol has been described using murine microglia, it can easily be adapted to human microglia, derived from induced pluripotent stem cells (iPSCs), depending on the research focus.^1^^,^^18^^,^^19^

### Synaptosome purity

While this protocol yields a synaptosome fraction enriched in synaptic markers, it is not entirely free from contamination by myelin and other cellular compartments. As a result, measurements performed using this preparation may not be entirely specific to synaptosomes. More stringent isolation protocols, which involve ultracentrifugation steps, can provide higher purity but may be less accessible due to specialized equipment requirement and longer processing times. To assess the purity of the preparation, validation using synaptic and non-synaptic markers (e.g., Western blot or immunostaining) is recommended to account for potential non-synaptic contributions to the observed results.

For pure synaptosome isolation, please follow ad hoc protocols.^20^

### Synaptosome uptake and degradation

The details of the protocol described here specifically refer to primary microglia cells. However, the protocol can be applied to other cell types, modifying the cargo concentration, and the timing of incubation to allow adequate cargo uptake. Timing of degradation following cargo washout can also be adjusted, based on the degradative dynamics of the cell type of interest. Furthermore, it is important to emphasize that the data obtained with this protocol pertain to an *in vitro* setting, where microglia are maintained in culture without other cells, which might influence microglial phagocytic capacity.

Another limitation of this protocol is that after the wash out of extracellular cargo, any phagocytosed material that has not yet reached the late endosome or lysosome, as well as membrane-bound material, will continue to be processed. As a result, the degradation phase will depend on how much cargo is still bound to microglia, particularly when assessing the early stages of phagocytosis. This may result in a paradoxical increase in the internalized signal, after medium washout, when performing live imaging.

Using a combination of fluorescently labeled and pHrodo-conjugated cargo, together with live imaging approaches, will provide a more comprehensive view of both the uptake and degradation processes.

### Moderate-throughput approach

We acknowledge that confocal imaging is not as high throughput approach. However, it offers significant advantages in assessing microglial phagocytosis. One of the key strengths of our protocol is the ability to track live microglial cells over extended periods, enabling dynamic real-time observation of phagocytosis. This capability allows for the analysis of cellular behaviors and the phagocytic process in action, something that is not possible with high-throughput methods like flow cytometry. Additionally, confocal imaging offers the spatial resolution needed to examine individual cells, revealing the variations in phagocytic activity across different cells.

This variability is important because it helps identify differences in how individual microglia respond, which can be overlooked in bulk population analyses.

Alternatively, flow cytometry could be used to assess phagocytosis and a few protocols have been described.^21^^,^^22^^,^^23^ This approach offers significant advantages, particularly in high-throughput analysis. It allows for rapid, quantitative assessment of fluorescent uptake across large cell populations, making it ideal for screening multiple conditions or samples. While it lacks the spatial and temporal resolution of confocal imaging, flow cytometry excels in providing quick, population-level data on phagocytic activity.

Furthermore, primary microglia can be time-consuming to work with due to the challenges of isolation and long culture periods. For high-throughput studies, cell lines offer a more efficient alternative (e.g. BV-2 murine microglial cell line), though they have several limitations. While easier to maintain and expand, cell lines do not fully mimic the behavior of primary microglia, particularly in terms of their response to the microenvironment, metabolism and phagocytic activity. Ideally, findings obtained using cell lines should always be validated in primary cultures, and finally *in vivo*.

## Troubleshooting

### Problem 1

Microglia don’t grow or die (Related to “before you begin, maintenance of culture, step 20”).

### Potential solution

If the flask appears empty or less than 50% confluent by DIV 14, the cell culture preparation is not successful and needs to be excluded.

• Not removing the meninges will induce significant fibroblast contamination due to their rapid proliferation rate under these culture conditions and prevent astrocyte growth. Using sharp forceps facilitates the pinching of the meninges, and efficient removal, leaving the brain unaffected.
• This could be due to a low density of cells when seeding. You can increase the density of cells, either by increasing the number of pups per T75 flask or using a smaller flask (T25).
• Cells don’t adhere to the T75 flask. Pre-coating with Poly-D lysine (0.1 mg/mL) for at least 1 h, at 37°C, helps to attach the cells to the flask. Waiting for 48 h before the complete change of media after plating (instead of 24 h) can also help. Remember to wash well (at least 3 times in water, as Poly-D-lysine is toxic to the cells).
• Some FBS lots can represent an issue for proper microglial growth. Make sure to take heat-inactivated or endotoxin-low FBS.
• Microglia ready to be harvested will pop out on top of the astrocytic layer (around day 10-14 in culture). They will appear as bright round cells. Make sure to collect the cells when you see that they start detaching (see Figure 1A).

### Problem 2

Low yield and low microglia density (Related to “before you begin, seeding, step 22”).

### Potential solution

This can come from excessive microglial death. Make sure to set the centrifuge to 350 x g (see “primary microglia culture”, step 26) in the collection step, as excessive speed and rotation will lead to cell death.

This can also come from too gentle (few cells detached) or too harsh tapping of the flask to detach microglia. Alternatively, place the flasks on an orbital shaker inside the incubator, for 1 h at 300 rpm.

Make sure to tap/shake strongly enough to detach the microglia from the astrocytic layer. After that, check under a microscope to assess the detachment. If not, continue the tapping/shaking for longer periods. After collection, check again under a microscope to verify that you don’t have any (or only a few) microglia remaining.

We suggest checking their morphology under the microscope 48 h after seeding, to ensure proper attachment and expected appearance of some processes. If too low in density, microglia will suffer and die, and they cannot be used for experiments.

### Problem 3

Microglia culture is not pure. Astrocytes, endothelial cells, fibroblasts/pericytes contamination (Related to “before you begin, seeding, step 22”).

### Potential solution

The purity of the isolated primary microglia can be assessed by immunofluorescence (e.g. staining against P2Y12R -microglia marker-, or IBA1 -microglia/macrophage marker-). If the purity is not optimal, astrocytes, endothelial cells or fibroblasts may be the cause of the contamination. In this case, it would be helpful to stain against GFAP, CD31 and PDGFRb, respectively.

Astrocyte contamination could be due to excessive tapping/shaking of the flask. Reducing the time of the shaking or the strength of the tapping may help improving microglia purity. Endothelial or pericytes contamination could be due to inappropriate meninges removal.

### Problem 4

Synaptosomes are not pHrodo-labeled (Related to “before you begin, synaptosome preparation and pHrodo conjugation, step 54”).

### Potential solution

Make sure that the sodium bicarbonate buffer is at the right pH (pH 8.4).

Synaptosomes preparation needs to be stored correctly and be thawed on ice. Make sure to resuspend the solution properly after thawing.

Prior to the experiment, mix a small amount of synaptosomes in an acidic solution and observe the signal under a fluorescent microscope.

### Problem 5

Microglia cells move out from the field of view during live-imaging recording (Related to “step-by-step method details, assessing synaptosome uptake in primary microglia by live imaging, step 26”).

### Potential solution

As microglia cells are highly mobile, they may move out from the field of view during the recording. Decreasing the magnification, either changing the objective or reducing the digital zoom may help. Consider acquiring a larger area of the plate tiling multiple fields of view. For the analysis, we recommend excluding the cells that move out from the field of view at later time points.

### Problem 6

No signal is observed during cargo uptake (Related to “step-by-step method details, assessing synaptosome uptake in primary microglia by live imaging, step 26”).

### Potential solution

Make sure that the cargo is well conjugated with the pHrodo (refer to problem 4).

Depending on the cargo, the optimal time for uptake and degradation should be optimized. Increase the duration of uptake if needed.

Especially for live-imaging, microglial stress could be the cause. Temperature, gas, and humidity should be optimal for proper microglial function.

Before starting the experiment, observe microglia morphology under a microscope and assess viability. They should be ramified.

For live imaging, assess motility. Microglia that stop moving may indicate cellular stress and potential death.

### Problem 7

No decrease in signal is observed during cargo degradation (Related to “step-by-step method details, assessing synaptosome degradation in primary microglia by live imaging, step 33”).

### Potential solution

This can be due to excessive cargo remaining in the well after the washes at the end of the uptake period, which may continuously be internalized within microglia. You can increase the number of washes.

Make sure to not “over” coat the wells, otherwise synaptosomes will attach to the bottom of the wells. We recommend a 1 h poly-D-lysine coating, followed by three washes with dH_2_O.

As for the uptake, especially for live-imaging, microglial stress could be the cause. Temperature, gas, and humidity should be optimal for proper microglial function.

Before starting the experiment, observe microglia morphology under a microscope and assess viability. They should be ramified.

For live imaging, assess motility. Microglia that stop moving may indicate cellular stress and potential death.

## Resource availability

### Lead contact

Further information and requests for resources and reagents should be directed to the lead contact, Prof. Rosa Chiara Paolicelli (rosachiara.paolicelli@unil.ch).

### Technical contact

For technical support, contact either Dr. Alessandro Matera (alessandro.matera@unil.ch) or Dr. Anne-Claire Compagnion (anne-claire.compagnion@unil.ch).

### Materials availability

This study did not generate new unique reagents.

### Data and code availability

This study did not generate any unique dataset or code.

## Acknowledgments

This work was supported by grants from an ERC StGrant (REMIND 804949), the Dementia Research Synapsis Foundation, and funding from UNIL to R.C.P.

## Author contributions

A.M. and A.-C.C. wrote the manuscript, which was edited and supervised by R.C.P. A.M. and A.-C.C. performed all the experiments. R.C.P. supervised the research.

## Declaration of interests

The authors declare no competing interests.
