# Supplementary material for: Protocol to assess engulfment and degradation of synaptosomes by murine microglia in vitro
Source: STAR Protoc. 2025 Jul 10;6(3):103936. doi: 10.1016/j.xpro.2025.103936 (PMC12274743; doi:10.1016/j.xpro.2025.103936)
Supplement: Document S1. Figure S1 [file mmc1.pdf]

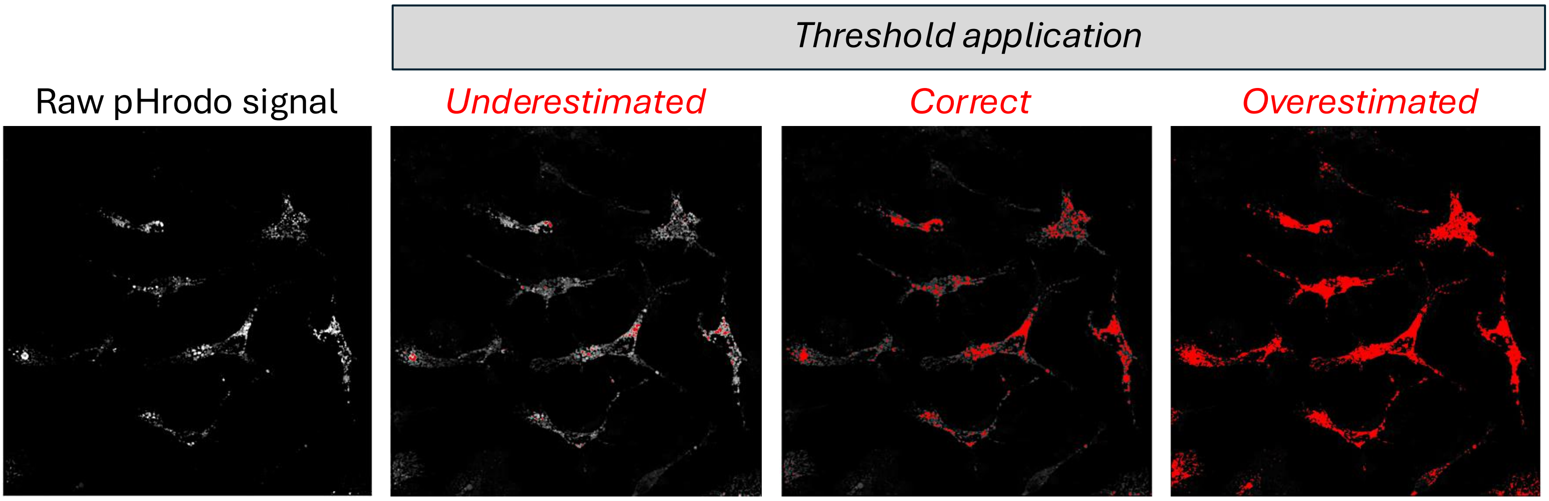

Suppl. Figure 1: Correct thresholding of pHrodo signal, related to "Step-by-step method details, step 37".
Representative images of optimal threshold settings.

Suppl. Figure 1
